# TLR3 Regulated Poly I:C-Induced Neutrophil Extracellular Traps and Acute Lung Injury Partly Through p38 MAP Kinase

**DOI:** 10.3389/fmicb.2018.03174

**Published:** 2018-12-21

**Authors:** Tingting Gan, Yonglin Yang, Fan Hu, Xichen Chen, Jiawei Zhou, Yan Li, Ying Xu, Huijuan Wang, Yu Chen, Mingshun Zhang

**Affiliations:** ^1^Department of Anesthesiology, Jiangsu Province Hospital, The First Affiliated Hospital of Nanjing Medical University, Nanjing, China; ^2^Department of Infectious Diseases, Nanjing First Hospital, Nanjing Medical University, Nanjing, China; ^3^NHC Key Laboratory of Antibody Technique, Nanjing Medical University, Nanjing, China; ^4^State Key Laboratory of Reproductive Medicine, Nanjing Medical University, Nanjing, China; ^5^Analysis Center, Nanjing Medical University, Nanjing, China; ^6^Nanjing University Medical School, The Affiliated Nanjing Drum Tower Hospital, Nanjing, China; ^7^Department of Immunology, Nanjing Medical University, Nanjing, China

**Keywords:** poly I:C, acute lung injury, neutrophil extracellular traps, p38, claudin-5

## Abstract

Acute lung injury (ALI) is the leading cause of morbidity and mortality in critically ill patients. Neutrophil extracellular traps (NETs) have been well documented in the ALI model of bacterial infection. In the present study, we demonstrated that poly I:C could induce pulmonary NETs. Upon poly I:C intratracheal inoculation, neutrophil infiltration in the bronchoalveolar lavage fluid (BALF) was significantly increased. Furthermore, the inflammatory cytokines IL-1β, IL-6, and TNF-α in the lung were also significantly elevated. Neutrophil depletion abolished NETs and decreased both neutrophil infiltration and IL-1β in the lung. As expected, DNase I, an inhibitor of MPO and NADPH, decreased pulmonary inflammation and NETs. Blocking of the poly I:C receptor TLR3 reduced lung inflammation and NETs. The MAPK kinase inhibitor p38 diminished the formation of NETs and restored the expression of the tight junction protein claudin-5 in the mouse lung when challenged with poly I:C. In summary, poly I:C induced the formation of pulmonary NETs and ALI, which may be associated with the activation of p38 MAPK and the decreased expression of claudin-5.

## Introduction

Acute lung injury (ALI) and acute respiratory distress syndrome (ARDS) are the leading causes of morbidity and mortality in critically ill patients (Butt et al., 2016). Neutrophils, which are orchestrators and key players of the innate immune response, play diverse roles in ALI and ARDS (Abraham, 2003; Zhou et al., 2012). Inflammatory cytokines and chemokines from these accumulated activated neutrophils, evoke and aggravate lung inflammation. For example, in the lung-protective ventilatory strategy, neutrophil quantification and pro-inflammatory cytokine concentrations were markedly reduced (Goodman et al., 2003). In contrast, the uncontrolled and lethal cytokine storm, which recruited neutrophils and other immune cells, was responsible for ALI in the deadly H5N1 influenza virus infection (Tisoncik et al., 2012). Furthermore, the influenza virus triggered the induction of IL-8 and other inflammatory cytokines from neutrophils (Wang et al., 2008). Neutrophil extracellular traps (NETs) consist of nuclear chromatin, hyper citrullinated histones, granular antimicrobial proteins and cytoplasmic proteins. In an ALI model of bacterial infection (Narayana Moorthy et al., 2013), LPS challenge (Tadie et al., 2013; Liu et al., 2016), transfusion (Hishizawa et al., 2009; Looney et al., 2009), and ventilation (Yildiz et al., 2015), NETs have been well documented, suggesting pivotal roles for NETs in ALI.

The human respiratory syncytial virus (RSV) induces NETs in the lungs, contributing to airway obstruction (Cortjens et al., 2016). The influenza virus infection recruits large amounts of neutrophils, leading to the development of NETs in the lung and contributing to ALI (Narasaraju et al., 2011). NETs may also be linked to the human immunodeficiency virus (HIV), which is detected by Toll-like receptor 7 (TLR7) and TLR8 in neutrophils and induces the generation of NETs (Saitoh et al., 2012). The RSV, influenza virus and HIV are single-stranded viruses. In one study, poly I:C, a synthetic analog of a double stranded RNA (dsRNA) virus, stimulated NETs in the liver (Jenne et al., 2013). Though poly I:C induced lung inflammation and impaired lung function in mice (Stowell et al., 2009), whether poly I:C could induce NETs in the lungs as well as their role in ALI, remain largely elusive.

TLR3 is a potent poly (I:C) receptor expressed in the lung epithelial cells (Bals and Hiemstra, 2004). Furthermore, poly (I:C) induces the elevated expression of TLR3 in the small airway epithelial cells (Ritter et al., 2005). Mitogen-activated protein kinase (MAPK) p38 is required for poly (I:C)/TLR3-mediated cytokine production (Pisegna et al., 2004). The lung barrier is largely controlled by tight junction proteins known as claudins (Schlingmann et al., 2015). Claudin-5, which is expressed by both the pulmonary epithelial and endothelial cells, is closely associated with acute lung injury (Chen et al., 2014).

In this study, we provided a detailed *in vivo* exploration of NETs in poly I:C-induced ALI, which was involved in the TLR3/p38 MAPK signaling pathway and the aberrant expression of claudin-5. Therefore, we hypothesized that NETs may be potential targets in the therapy of dsRNA virus-associated ALI.

## Materials and Methods

### Experimental Animals

Female C57BL/6J mice that were 6–8 weeks of age and free of specific pathogens, were obtained from the College of Veterinary Medicine Yangzhou University (Yangzhou, China). All experimental animals used in this study were maintained under a protocol 1709011 and approved by the Institutional Animal Care and Use Committee of the Nanjing Medical University. All methods were performed in accordance with the relevant guidelines and regulations.

### Acute Lung Injury Model and Treatment

The acute lung injury model was established as described previously (Aeffner et al., 2011). Briefly, the mice were anesthetized with an intraperitoneal (IP) injection of a mixture of ketamine (200 mg/kg) and xylazine (10 mg/kg). After exposing the trachea, a trimmed sterile 31-gauge needle was inserted into the tracheal lumen. Poly I:C (InvivoGen, CA, United States) diluted in endotoxin-free PBS (SCBT, sc-286634) was intratracheally (IT.) injected at a dose of 2.5 mg/kg in 50 μl PBS, followed by 100 μl air to ensure the full distribution of poly I:C into the lung. PBS was only instilled as an ALI model control.

To deplete neutrophils, anti-Ly6G (clone 1A8, Bio X Cell, NH, United States) or isotype control Ab at a dose of 200 μg in 200 μl PBS, was administered via the tail vein, 24 h before the establishment of the ALI model (Daley et al., 2008). To treat ALI mice, a DNase I (Roche, 11284932001), MPO inhibitor (4-ABH, sigma, A41909), NADPH inhibitor (Diphenyliodonium chloride, sigma, 43088), TLR3 inhibitor (Merck, 614310) or p38 inhibitor (SB203580, Selleck) was dissolved in DMSO and administered via an IP injection, 2 h before the inoculation of poly I:C. DNase I was given at 5 mg/kg, MPO inhibitor at 50 mg/kg, TLR3 inhibitor at 50 mg/kg, NADPH inhibitor at 10 mg/kg, and p38 inhibitor at 50 mg/kg. DMSO administration was set as the treatment control.

### Analysis of BALF Samples

Four hours after the poly I:C or PBS challenge, the mice were anesthetized via an IP injection of a mixture of ketamine (200 mg/kg) and xylazine (10 mg/kg). A catheter was inserted into the trachea by way of an injected opening located in the cervical part, and the airways were washed three times with a total of 1.0 ml PBS. The pooled bronchoalveolar lavage fluid (BALF) was centrifuged at 1000 *g* for 5 min at 4°C, and the total cells were quantified with a hemocytometer, by two independent blinded investigators. Furthermore, the supernatant was collected to measure the quantity of protein using the Bradford Protein Assay Kit (Beyotime, P0006). The total cell counts and protein concentration in the BALF from the PBS control, isotype antibody control and DMSO control were greatly similar. Therefore, the data for the cell counts and protein concentration were pooled.

### Flow Cytometry Analysis

Next, the cells in the BALF were stained with fluorescent antibodies to quantify neutrophils, macrophages and lymphocytes. Briefly, the cells in the BALF were blocked with anti-CD16/CD32 (BioLegend, 101302) to reduce non-specific binding at 4°C for 10 min. Anti-Gr-1 conjugated with APC (BioLegend, 108412), anti-F4/80 conjugated with FITC (eBioscience, 11-4801-81), and anti-CD4 conjugated with PE (eBioscience, 12-0042-85) were added to label neutrophils, macrophages and CD4^+^ lymphocytes at 37°C for 30 min in darkness. Finally, the staining tubes were centrifuged to remove the supernatant, and the cells were resuspended in 500 μl PBS and analyzed in the BD FACSCalibur. All FACS data were analyzed using the FlowJo V10.

### Histopathology Imaging

After the BALF was collected, PBS was pumped into the right ventricle to clear the blood in the pulmonary vasculature. Then, the upper right lung lobe was removed and fixed in 10% neutral buffered formalin for 24 h; then, the specimens were dehydrated and embedded in paraffin. To carry out a histological examination, 5 μm sections of fixed embedded tissues were cut on a Leica model 2165 rotary microtome (Leica, Nussloch, Germany) and stained with hematoxylin and eosin. Histological analyses were performed by two independent pathologists blinded to the treatment groups.

### RNA Preparation and Quantitative RT-PCR

After the upper lobe was removed for histopathology, the total RNA was purified from the residual right lung lobes using a TRIzol reagent (Invitrogen Life Technologies) and reverse transcribed using the Superscript II (Takara) to obtain the cDNA. Real-time PCR using the SYBR^®^ Premix Ex Taq^TM^ (Takara, Tli RNaseH Plus) and the PrimeScript^TM^ RT Master Mix (Takara, Perfect Real Time) was performed on the cDNA samples. The amplification conditions were 1 cycle at 50°C for 2 min, 1 cycle at 95°C for 30 s, followed by 40 cycles of thermal cycling at 95°C for 5 s and 60°C for 45 s.

Primer pair DNA sequences are shown as follows: (1) IL-1β, upper: 5′-AGCTCTCCACCTCAATGGA-3′; lower: 5′-TTGCTTGGGATCCACACTCT-3′; (2) IL-6, upper: 5′-GACTGATGCTGGTGACAACC-3′; lower: 5′-AGACAGGTCTGTTGGGAGTG-3′; (3) TNF-α, upper: 5′-GGTGAGGCAGCAAGAGATTG-3′, lower: 5′-GAGCAGCAGGTTTCAGGATG-3′. β-actin was used as an internal control using the following primer sequences: upper: 5′-ATGTTTGAGACCTTCAACAC-3′, lower: 5′-CACGTCACACTTCATGATGG-3′. Quantitative PCR assays were conducted in triplicate for each sample and performed using the 2-ΔΔCt method. Data were expressed as an n-fold difference relative to the expression of inner housekeeping gene β-actin. Blank controls lacking a template or without a reverse transcriptase, were also performed.

### Fluorescence Microscopy

The left lung lobe was removed and immobilized with OCT (Sakura Finetek) at -80°C for at least 24 h. Frozen tissue blocks were cut on a cryostat microtome, and 5 μm sections were placed on coated glass slides. The tissue sections were fixed in cold acetone and rehydrated in PBS. After gently washing in PBS three times, the slides were blocked with 5% goat serum (Gibco, 16210-064) for 30 min at 37°C to reduce non-specific binding. After blocking, the sections were washed with PBS and stained with myeloperoxidase antibody (Abcam, ab90810) and anti-Histone H3 (citrulline R2 + R8 + R17) (Abcam, ab5103) at 4°C overnight in darkness. After staining, the sections were gently washed with PBS, followed by incubation with the secondary antibodies: Alexa Fluor 647-Anti-mouse IgG (Life Technology, A32728) or Alexa Fluor 555-Anti-rabbit IgG (Life Technology, A21428) at 37°C for 60 min in darkness. After washing in PBS to remove the unbound fluorescent antibody, the sections were incubated with Sytox Green (Thermo, s7020) diluted in PBS (1:2000) at 37°C for 15 min. The sections were rinsed again and mounted with glycerol. The frozen sections were recorded using confocal microscopy (Carl Zeiss LSM710). Positive fluorescence (MPO staining and citrullinated histone 3 staining) was calculated with the ImageJ software. The data were expressed as the percentage of the area in each field of view, covered by positive fluorescent staining.

Neutrophil extracellular traps in the BALF were quantified according to the manufacturers’ recommendations for the NETs Assay Kit (NO. 601010, Cayman) with modification. Briefly, the selective substrate was added to the BALF and quantified at 405 nm. The elastase activity in each BALF sample was calculated according to the NET standards. Instead, elastase activity could be indicative of NETs or neutrophil necrosis.

### Western Blotting

The lower right lung lobes were removed and stored in liquid nitrogen for protein analysis. Briefly, the lung tissues were lysed on ice in a RIPA (P0013B, Beyotime) buffer containing 1 mM PMSF (Beyotime). After sonication, the protein concentration in the supernatants was measured using a bicinchoninic acid protein assay kit (Beyotime). Equal amounts of protein were loaded in 10% SDS–PAGE, and then the proteins were transferred to polyvinylidene fluoride membranes and blocked in 5% bovine serum albumin for 1 h at room temperature. After rinsing in PBST 5 times, the membranes were incubated with each primary antibody overnight at 4°C: the anti-claudin 5 (Abcam, ab15106), the phosphor-MAPK family Antibody sampler kit (cell-signaling technology, #9910) the MAPK family Antibody sampler kit (cell-signaling technology, #9926), and β-actin (cell-signaling technology, #4970). After washing with PBST, the membranes were finally incubated with the appropriate secondary antibody for 1 h at room temperature. Protein bands were detected using an ECL High-Signal reagent (Thermo). The band intensity was quantified using the ImageJ software.

### Statistical Analysis

The experiments were repeated at least three times with consistent results. The significant difference between experimental groups and the control group was performed using the Student’s *t*-test with the SPSS software (SPSS, Chicago, IL, United States). *P* < 0.05 was considered statistically significant. All data are expressed as the mean ± SEM.

## Results

### Poly I:C Induced NETS in ALI

**FIGURE 1:**
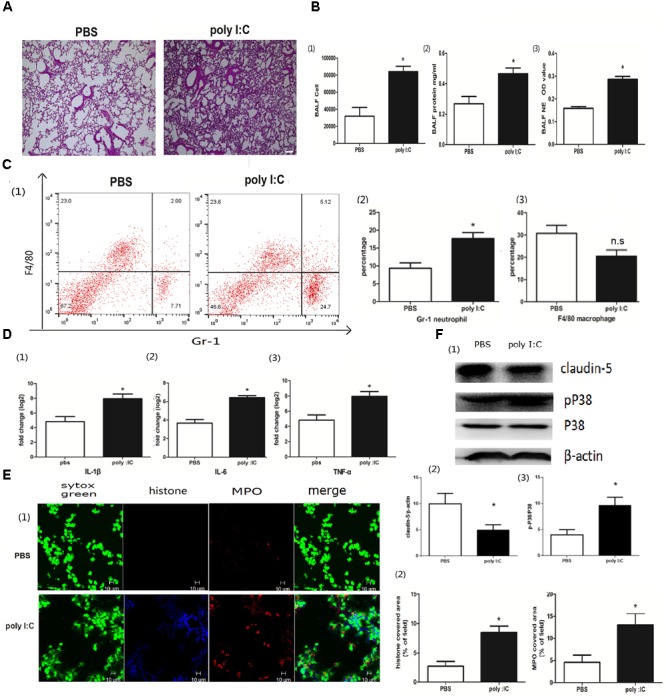
Poly I:C-induced inflammation and NETs in mice with ALI. **(A)** Infiltration of inflammatory cells and interstitial edema in the lung lobes from poly I:C-stimulated mice, bar = 100 μm; **(B-1)** cells were counted in the BALF, ^∗^*p* < 0.05; **(B-2)** protein concentration in the BALF, ^∗^*p* < 0.05; **(B-3)** neutrophil elastase (NE) activity was evaluated in the BALF, ^∗^*p* < 0.05; **(C)** flow cytometry analysis of the BALF cells, ^∗^*p* < 0.05; **(D)** inflammatory cytokines IL-1β, IL-6, and TNF-α in the lung were quantified using qRT-PCR, ^∗^*p* < 0.05; **(E)** NETs were observed and quantified in lung tissues from mice administered poly I:C. **(F)** Claudin-5 and p38 MAPK were measured in lung tissues using Western-blot.

To determine whether poly I:C could induce NETs in the lung, we first inoculated poly I:C directly into the lung via the trachea. After poly I:C instillation, inflammation occurred in the lung, which was characterized by the infiltration of inflammatory cells and interstitial edema (Figure 1A). In accordance with the elevated lung inflammation, The BALF cells and proteins were significantly increased. Moreover, elastase activity was increased in the BALF for the mice inoculated with poly I:C, suggesting that poly I:C may induce NETs in the lung (Figure 1B). A flow cytometry analysis indicated that neutrophils, but not macrophages, were significantly increased in the BALF (Figure 1C). Additionally, the addition of poly I:C led to an increase in the inflammatory cytokines IL-1, IL-6, and TNF-α in the lung (Figure 1D). In the lung tissues from poly I:C treated mice, the intensities of MPO and citrullinated histone 3 (markers of neutrophils and NETs, respectively), were significantly increased (Figure 1E). The tight junction protein claudin-5, which may be responsible for ALI, was decreased in the lung tissues for poly I:C treated mice. Additionally, p38 MAPK was activated in poly I:C treated lung tissues, suggesting that the p38 MAPK pathway may be associated with poly I:C induced lung injury (Figure 1F). In summary, poly I:C induced NETs in ALI may be associated with elevated inflammatory cytokines, aberrant claudin-5 and the p38 MAPK pathway.

### Neutrophil Depletion Reduced NETs and ALI

**FIGURE 2:**
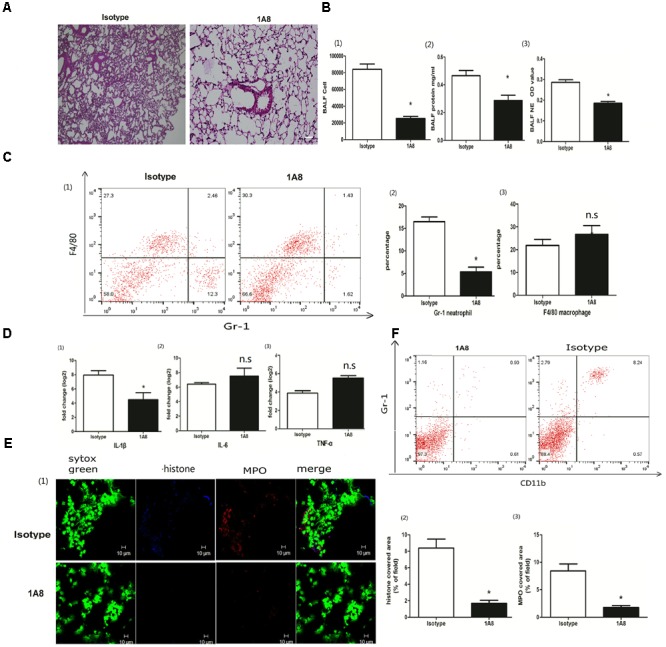
Neutrophil depletion reduced NETs and ALI. **(A)** Infiltration of inflammatory cells and interstitial edema in the lung lobe were reduced in the ALI model mice with neutrophil depletion, bar = 100 μm; **(B-1)** cells were counted in the BALF, ^∗^*p* < 0.05; **(B-2)** protein concentration in the BALF, ^∗^*p* < 0.05; **(B-3)** neutrophil elastase (NE) activity was evaluated in the BALF, ^∗^*p* < 0.05; **(C)** flow cytometry analysis of the BALF cells, ^∗^*p* < 0.05; **(D)** inflammatory cytokines IL-1β, IL-6, and TNF-α in the lung were quantified using qRT-PCR, ^∗^*p* < 0.05; **(E)** NETs were observed and quantified in lung tissues from mice administered poly I:C and 1A8 antibody. **(F)** Effects of 1A8 on neutrophil depletion were validated by flow cytometry.

Extracellular traps can be released not only by neutrophils but also by macrophages (Boe et al., 2015). To better clarify the roles of neutrophils in the extracellular traps and ALI, we injected a specific Ly6G antibody (1A8) via the tail vein to deplete neutrophils (Sun et al., 2016; Zhang et al., 2016). Neutrophil depletion ameliorated lung inflammation, resulting in less infiltration of inflammatory cells and less interstitial edema (Figure 2A). The BALF cells and proteins, which are hallmarks of the severity of acute lung injury, were also significantly decreased in the mice with neutrophil depletion (Figure 2B). Elastase activity, which indicates the formation of NETs, was significantly reduced in the mice with neutrophil depletion (Figure 2B). The inflammatory cytokine IL-1β but not IL-6 or TNF-α was also significantly decreased in the mice with neutrophil depletion (Figures 2C,D). Moreover, 1A8 treatment almost abolished the extracellular traps, as indicated by the nearly undetectable levels of MPO and citrullinated histone 3 (Figure 2E). The role of 1A8 in neutrophil depletion is validated in Figure 2F. In summary, neutrophils rather than macrophages were the major producer of extracellular traps in poly I:C induced acute lung injury.

### DNase Protected the Lung From Poly I:C-Induced NETs

**FIGURE 3:**
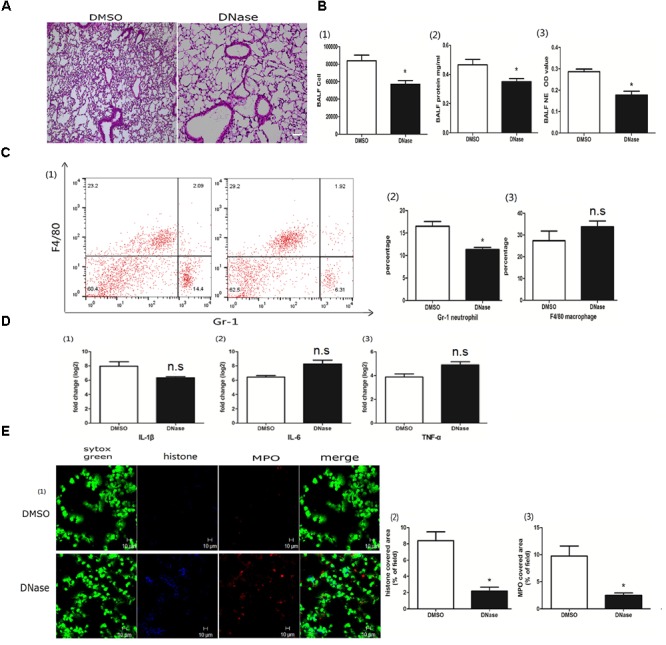
DNase protected the lung from poly I:C-induced NETs. **(A)** Infiltration of inflammatory cells and interstitial edema in the lung lobe were reduced in the ALI model mice upon DNase treatment, bar = 100 μm; **(B-1)** cells were counted in the BALF, ^∗^*p* < 0.05; **(B-2)** protein concentration in the BALF, ^∗^*p* < 0.05; **(B-3)** neutrophil elastase (NE) activity was evaluated in the BALF, ^∗^*p* < 0.05; **(C)** flow cytometry analysis of the BALF cells, ^∗^*p* < 0.05; **(D)** inflammatory cytokines IL-1β, IL-6, and TNF-α in the lung were quantified using qRT-PCR, ^∗^*p* < 0.05; **(E)** NETs were observed and quantified in lung tissues from mice administered poly I:C and DNase.

DNA is the scaffold of the NET (Zawrotniak and Rapala-Kozik, 2013). To better explore the roles of NETs in acute lung injury, we treated the mice with DNase I to degrade poly I:C-induced NETs. DNase administration reduced lung inflammation (Figure 3A). Infiltrated cells, proteins and NET formation in the BALF were also significantly reduced in the DNase I-treated mice (Figure 3B). Additionally, neutrophils in the BALF were significantly decreased in the DNase I-treated mice (Figure 3C). Interestingly, DNase I treatment played a non-significant role in the production of inflammatory cytokines in the lung tissue (Figure 3D). However, DNase I treatment significantly reduced the formation of NETs (Figure 3E), further implying that poly I:C-induced NETs may contribute to acute lung injury.

### MPO Contributed to Poly I:C Induced NETs

**FIGURE 4:**
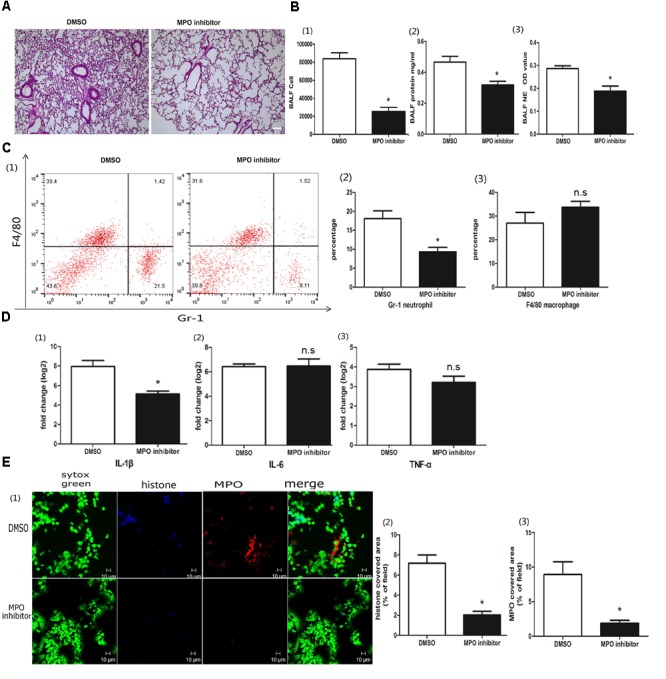
MPO contributed to poly I:C induced NETs. **(A)** Infiltration of inflammatory cells and interstitial edema in the lung lobe were decreased in the ALI mice treated with an MPO inhibitor, bar = 100 μm; **(B-1)** cells were counted in the BALF, ^∗^*p* < 0.05; **(B-2)** protein concentration in the BALF, ^∗^*p* < 0.05; **(B-3)** neutrophil elastase (NE) activity was evaluated in the BALF, ^∗^*p* < 0.05; **(C)** flow cytometry analysis of the BALF cells, ^∗^*p* < 0.05; **(D)** inflammatory cytokines IL-1β, IL-6, and TNF-α in the lung were quantified using qRT-PCR, ^∗^*p* < 0.05; **(E)** NETs were observed and quantified in lung tissues from mice administered poly I:C and an MPO inhibitor.

MPO, the peroxidase enzyme in neutrophils, was required for NET formation in a *Candida albicans* infection (Metzler et al., 2011). To investigate whether MPO was involved in poly I:C-induced NETs in acute lung jury, we treated the mice with the MPO inhibitor 4-aminobenzoic hydrazide (4-ABH). Interestingly, 4-ABH suppressed the infiltration of inflammatory cells in the lung (Figure 4A). The BALF cells, proteins and NETs were all significantly reduced in mice upon 4-ABH administration (Figure 4B). Flow cytometry analysis revealed that MPO inhibition resulted in reduced neutrophil infiltration in the BALF (Figure 4C). IL-1β in the lung was significantly decreased in the 4-ABH treated mice (Figure 4D). Furthermore, 4-ABH almost eradicated the NETs (Figure 4E), suggesting the pivotal role of MPO in poly I:C-induced NETs and ALI.

### NADPH Was Associated With the Poly I:C-Induced NETs

**FIGURE 5:**
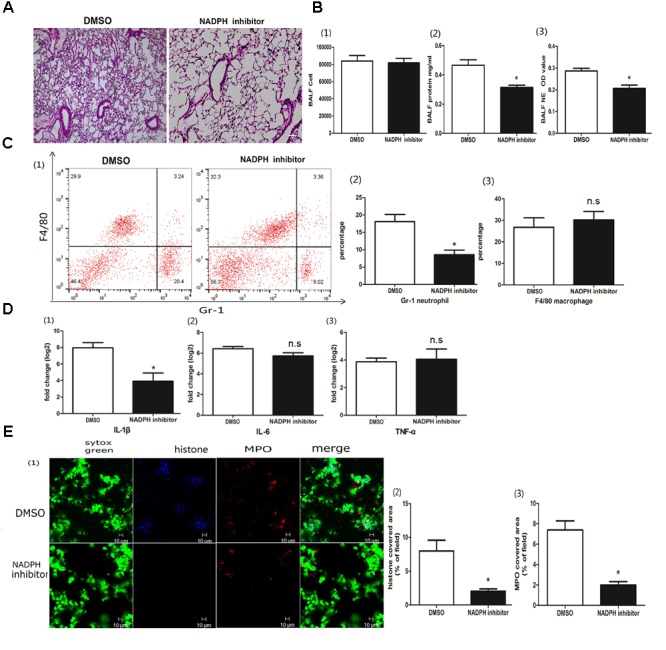
NADPH was associated with poly I:C-induced NETs. **(A)** Infiltration of inflammatory cells and interstitial edema in the lung lobe were inhibited in the ALI mice treated with an NADPH inhibitor, bar = 100 μm; **(B-1)** cells were counted in the BALF, ^∗^*p* < 0.05; **(B-2)** protein concentration in the BALF, ^∗^*p* < 0.05; **(B-3)** neutrophil elastase (NE) activity was were evaluated in the BALF, ^∗^*p* < 0.05; **(C)** flow cytometry analysis of the BALF cells, ^∗^*p* < 0.05; **(D)** inflammatory cytokines IL-1β, IL-6, and TNF-α in the lung were quantified using qRT-PCR, ^∗^*p* < 0.05; **(E)** NETs were observed and quantified in the lung tissues from mice administered poly I:C and an NADPH inhibitor.

The role of NADPH in the NETs was subject to the stimulus, and, previously, either NADPH-dependent or -independent NETs were reported (Marcos et al., 2010; Parker et al., 2012). To better clarify whether poly I:C-induced NETs relied on NADPH, we used the NADPH oxidase inhibitor diphenyliodonium chloride (DPI) to block the enzyme. With DPI administration, we observed less infiltration of inflammatory cells in the lung (Figure 5A). Although the quantification of inflammatory cells in the BALF was comparable, protein concentration and NETs in the BALF were significantly decreased (Figure 5B). Again, DPI significantly reduced neutrophils infiltration and IL-1β in the lung (Figures 5C,D). More importantly, DPI reduced the formation of NETs (Figure 5E), suggesting that the role of poly I:C-induced NETs in ALI was NADPH dependent.

### The Role of Poly I:C-Induced NETs in ALI Was TLR3 Dependent

**FIGURE 6:**
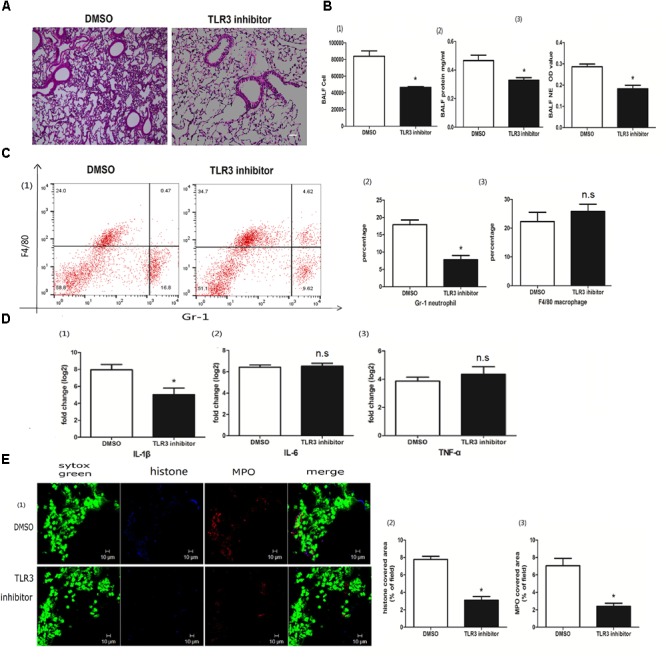
The role of poly I:C-induced NETs in ALI was TLR3 dependent. **(A)** Infiltration of inflammatory cells and interstitial edema in the lung lobe were reduced in the ALI mice treated with the TLR3 inhibitor, bar = 100 μm; **(B-1)** cells were counted in the BALF, ^∗^*p* < 0.05; **(B-2)** protein concentration in the BALF, ^∗^*p* < 0.05; **(B-3)** neutrophil elastase (NE) activity was evaluated in the BALF, ^∗^*p* < 0.05; **(C)** flow cytometry analysis of the BALF cells, ^∗^*p* < 0.05; **(D)** inflammatory cytokines IL-1β, IL-6, and TNF-α in the lung were quantified using qRT-PCR, ^∗^*p* < 0.05; **(E)** NETs were observed and quantified in lung tissues from mice administered poly I:C and a TLR3 inhibitor.

Toll-like receptor 3 (TLR3) is an intracellular poly I:C receptor (Prince et al., 2011). Unlike human neutrophils, mouse neutrophils express TLR3 (Applequist et al., 2002). To clarify whether the role of poly I:C-induced NETs in ALI was TLR3 dependent, we used a TLR3 inhibitor to suppress the recognition of TLR3 with poly I:C. The TLR3 inhibitor decreased not only lung inflammation (Figure 6A), but also cells and proteins in the BALF (Figure 6B), suggesting that poly I:C-induced ALI depends on TLR3. In line with the amelioration of ALI, NET formation in the BALF was almost eradicated upon TLR3 inhibitor administration (Figure 6B). Upon TLR3 blocking, the levels of neutrophil infiltration in the BALF (Figure 6C) and IL-1β in the lung (Figure 6D) were significantly decreased. Immunofluorescence microscopy showed that TLR3 inhibitors also significantly reduced NET formation in the lung (Figure 6E). Altogether, these data indicate that TLR3 is required for the role of poly I:C-induced NETs in ALI.

### p38 Regulated the Poly I:C-Induced NETs and ALI

**FIGURE 7:**
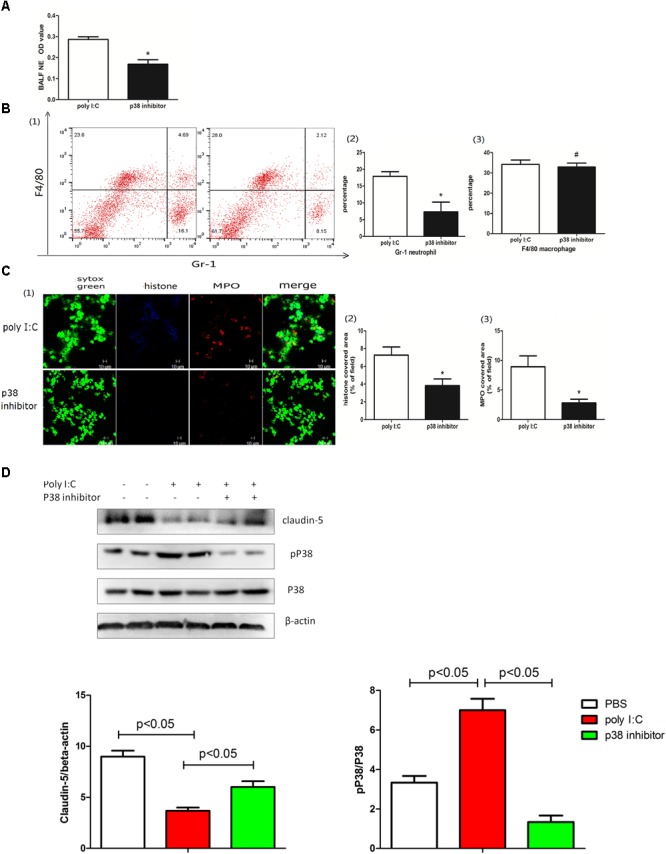
p38 regulated poly I:C-induced NETs and ALI. **(A)** neutrophil elastase (NE) activity was evaluated in the BALF, ^∗^*p* < 0.05; **(B)** flow cytometry analysis of the BALF cells, ^∗^*p* < 0.05 and ^#^*p* > 0.05; **(C)** NETs were observed and quantified in lung tissues from mice administered poly I:C and a p38 inhibitor. **(D)** Claudin-5 and p38 were measured in lung tissues via Western-blot; ^∗^*p* < 0.05.

Poly I:C bound with TLR3 evokes a cascade reaction, including the MAPK signaling pathway activation (Kawai and Akira, 2010). Moreover, the MAPK pathway was also shown to be required for NETs (Hakkim et al., 2011). To further explore whether p38 MAPK kinase contributes to poly I:C-induced pulmonary NETs, mice were treated with the p38 inhibitor SB203580. As shown in Figure 7A, the p38 inhibitor SB203580 decreased the production of NETs in the BALF. Furthermore, the p38 inhibitor reduced neutrophil infiltration in the BALF (Figure 7B) and suppressed NETs in the lung (Figure 7C). Interestingly, the p38 inhibitor partially recovered the expression of claudin-5 (Figure 7D), highlighting that p38 and claudin-5 might be potential targets in poly I:C-induced NETs and ALI.

## Discussion

As a critical illness syndrome, ALI is characterized by acute hypoxemic respiratory failure with the presence of bilateral diffuse pulmonary infiltrates (Wheeler and Bernard, 2007). Viral infections rank as top etiology of ALI in intensive care units (Erickson et al., 2007). More importantly, influenza virus (H1N1 (Narasaraju et al., 2011), H5N1 (Sun et al., 2012), H9N2 (Li et al., 2016)) and severe acute respiratory syndrome (SARS) virus (Kuba et al., 2005) might even induce deadly ALI and ARDS. It is not clear if viral replication or viral components (nucleic acids or proteins) are responsible for ALI and ARDS. In this study, we observed that poly I:C, a double-stranded RNA virus mimic, induced the infiltration of inflammatory cells in the lung, leading to ALI. Furthermore, extracellular diffuse DNA that was overlaid with MPO and citrullinated histone 3 (Wang et al., 2009) was recorded, suggesting a pulmonary NET formation. It is important to note that citrullinated histone 3 was not always perfectly overlaid with MPO. Citrullinated histone 3 has also been observed in the TNF-α associated inflammation (Sharma et al., 2012). MPO was most abundant in the neutrophils but was also present in monocytes and some populations of macrophages (McMillen et al., 2005). Collectively, we postulated that double-stranded viruses may evoke NETs via nucleic acids.

The Ly6G antibody 1A8 was more specific than the RB6-8C5, which recognized Ly6G on neutrophils and Ly6C on monocytes and macrophages; therefore, the latter antibody depleted neutrophils only (Daley et al., 2008). In this study, neutrophil depletion with 1A8 abolished NETs, confirming that the extracellular trap was released from neutrophils. In addition, DNA was the scaffold of NETs. DNase I, which degrades DNA, was indicated to be effective at eradicating NETs *in vivo* (Jenne et al., 2013). Different from Shuai Liu, who instilled DNase I into the trachea (Liu et al., 2016), we injected DNase I via the tail vein to break pulmonary NETs. Due to the anatomy of the lung, tracheal instillation with excessive liquid and air might cause pulmonary edema and emphysema, thus aggravating (rather than alleviating) acute lung injury. DNase I–coated nanoparticles effectively disrupted pulmonary NETs induced by cancer cells (Park et al., 2016), and they might be powerful tools for the treatment of poly I:C- or virus-induced ALI. In the ALI model, lung MPO was significantly correlated with intrapulmonary neutrophil diapedesis (Goldblum et al., 1985). It was shown that MPO is required for NET formation (Metzler et al., 2011). An MPO inhibitor suppressed NET formation and alleviated lung injury, suggesting that the MPO enzyme may be required for the development of poly I:C-induced NETs and ALI. The role of NADPH oxidase in NET formation is controversial. Furthermore, an NADPH inhibitor decreased both poly I:C-induced NET formation and ALI. Total leukocyte infiltration into the BALF, however, was not decreased upon NADPH inhibition. It was clear from the flow cytometry analysis that neutrophils were significantly reduced, and macrophages were slightly increased, which may explain the comparable diapedesis in the BALF for mice treated with or without the NADPH inhibitor.

TLR3, an intracellular receptor for viral RNA, is expressed in diverse cells. Therefore, we hypothesized that mouse neutrophils may directly recognize poly I:C via TLR3. Alternatively, epithelial cells expressing TLR3 rather than neutrophils might be a direct essential responder in TLR3 blocking (Murray et al., 2008). p38 MAPK is one of the downstream molecules of the TLR3 signaling pathway (Berube et al., 2009). In poly I:C-induced ALI, p38 MAPK was activated. Moreover, a p38 inhibitor reduced the formation of NETs and increased the expression of the tight junction protein claudin-5. In LPS-induced ALI, the roles of p38 and its inhibitor were controversial (Arcaroli et al., 2001; Liu et al., 2008). In swine influenza virus-induced ALI, p38 inhibition ameliorated the infiltration of inflammatory cells into the lung (Wei et al., 2014), which was in line with our observations. Of note, NETs may also be beneficial in pathogen killing (Brinkmann and Zychlinsky, 2007) and the resolution of inflammation by degrading inflammatory cytokines (Schauer et al., 2014). Therefore, the roles of NETs in poly I:C-induced ALI may warrant further investigation.

In conclusion, the present study provided direct evidence that poly I:C induced the formation of NETs in ALI. Neutrophils depletion or DNase reduced NETs, thereby alleviating the acute lung injury. Moreover, MPO and NADPH may be involved with poly I:C induced NETs in the lung. TLR3 and p38, the downstream signal molecules of TLR3, are at least partially responsible for poly I:C-induced NETs and ALI.

## Availability of Data and Materials

Please contact authors for data requests.

## Ethics Statement

This study was approved by the Institutional Animal Care and Use Committee of the Nanjing Medical University.

## Author Contributions

YC and MZ designed the experiments. TG and YY performed the animal experiments. FH and XC performed the confocal microscopy experiments. JZ and YL verified the pathological results. YX and HW edited and revised the manuscript. TG and MZ drafted the paper.

## Conflict of Interest Statement

The authors declare that the research was conducted in the absence of any commercial or financial relationships that could be construed as a potential conflict of interest.

## Abbreviations

4-ABH: 4-aminobenzoic hydrazide
ALI: acute lung injury
ARDS: acute respiratory distress syndrome
BALF: bronchoalveolar lavage fluid
DPI: diphenyliodonium chloride
IT: intratracheally
MPO: myeloperoxidase
NETs: neutrophil extracellular traps
SARS: severe acute respiratory syndrome
